# Supplementary material for: Computer vision detects covert voluntary facial movements in unresponsive brain injury patients
Source: Commun Med (Lond). 2025 Aug 20;5:361. doi: 10.1038/s43856-025-01042-y (PMC12368019; doi:10.1038/s43856-025-01042-y)
Supplement: Supplementary file 2 — Supplementary Information [file 43856_2025_1042_MOESM2_ESM.pdf]

Supplementary Methods

Criteria for Analyzable Videos

Analyzable videos did not have artifacts (doctor/clinician enters the field of view, bed/camera movement, and change in room lighting). The "Stick out your tongue" and "Show me a smile" videos were also not analyzable if they contained endotracheal tubes or securement devices that obstructed the mouth from view.

End of Study Participation

Once a patient reached one of the following endpoints, SeeMe recordings for that patient ceased.

- CRS-R Auditory subscore > 2 for five consecutive days.
- Deceased, terminally extubated, or placed on palliative care.
- Discharged from the hospital.
- The patient's LAR withdrew the patient from the study.
- Unable to stay in the frame of the video for more than ten recording sessions due to agitation or spontaneous movements.
- Was enrolled in the study for more than 60 days without a SeeMe positive result.

Data Processing Pipeline

SeeMe quantifies facial movements in response to verbal commands in ABI patients by vector field analysis. The algorithm tags individual facial pores (resolution ~0.2 mm) and tracks their movements in response to the presented stimulus. The data collection and video processing pipeline is as follows:

1. The auditory stimulation trial recorded videos were loaded into a Python-based video processing pipeline.
2. Timestamps of each command were automatically generated via auditory signal cross-correlation using the original auditory stimulation audio recordings for reference.
3. We extracted each video frame-by-frame.

4. A popular pre-trained deep learning model, "Mediapipe," was used to identify 478 facial landmarks, generating a "face mesh" on each frame (Supplementary Figure 6).[1]
5. We selected the landmarks comprising the subject's facial boundary, forming a mask. This mask was applied to each frame to isolate the subject's face.
6. We computed the displacement of each pixel area between that frame and the masked reference frame.[2] The reference frame was set as the frame located 10 seconds before the command presentation. Each pixel's displacement area formed a heatmap.
7. Next, we selected pixels and defined a specific region of interest (ROI) corresponding to the facial regions associated with each auditory stimulation type. The ROI for the "Stick out your tongue" and "Show me a smile" commands was 40 landmarks around the mouth area (Supplementary Figure 6). The "Open your eyes" command ROI was 158 landmarks around the eye area. Then, we summed the displacements of the landmarks in the ROI to represent the patient's response to the command in that frame.
8. We generated a time series for each video, with the x-axis representing the frame number (time) and the y-axis representing the ROI facial displacement/motion summation, representing the subject's facial response to the commands. Then, we used a moving average and low-pass filter to remove noise and movement caused by breathing that could increase the signal-to-noise ratio (SNR) (examples shown in Figure 2B and Supplementary Figure 1C).
9. We evaluated each trial by comparing the subject's facial motion between the 10 seconds before the command (pre-command) and the 20 seconds after each command presentation (post-command).
10. Afterward, we conducted a one-sided Kolmogorov-Smirnov (KS) test to assess the magnitude of the difference and whether the post-command response significantly differed from the pre-command response. We also used the maximum value of the post-command response to define the response magnitude (post-peak value).

11. We repeated the above steps for all ABI patients and healthy subjects. We obtained the optimum thresholds for KS stats and post-peak value (see *Kolmogorov-Smirnov and Post Peak Value*), which was 0.1 KS distance and 400 pixels. A trial is predicted SeeMe+ if its KS stats > 0.1 and post-peak > 400. As a positive control, we compared techniques in the healthy cohort. In the positive controls, blinded raters and SeeMe were almost 100% in agreement (Supplementary Figure 7, center columns of left and right panels). As a negative control, we limited the analysis to patients with CRS-R of ≤ 1, who we assumed to be deeply unconscious, and we predicted that there would be no detectable movement. This analysis showed that SeeMe detected almost no movements in those negative control patients**.**
12. A patient's command following was said to be detected by SeeMe if that particular command had ≥ 3/10 SeeMe+ trials. This is equivalent to a SeeMe+ video.

Kolmogorov-Smirnov and Post Peak Value

We used the recordings obtained from the healthy control subjects - where we know they are responding to every command - and the deeply comatose, motionless patients whose daily CRS-R was ≤ 1 - where we assumed they are not responding to any commands - to choose two thresholds (of note, there were no usable mouth videos with CRS-R = 0, so we used CRSR = 1 for this population). These thresholds were defined such that an ABI patient would be considered responding to a command if their KS stats and post-peak value in that trial were larger than the two thresholds. The thresholds were chosen by maximizing the prediction accuracy of SeeMe in healthy controls and in deeply comatose, motionless patients (for eye movement, see Supplementary Figure 8). Although using an amplitude threshold of 500 pixels (red triangle) has a higher prediction accuracy in motionless patients, it prevented SeeMe from detecting weak responses. Using 400 pixels (green triangle) still yielded accuracy similar to the 500 thresholds in healthy and motionless patients while simultaneously capturing weak responses. This caused us to choose a 0.1 KS distance and 400 pixels as the thresholds for SeeMe to detect stimulus-evoked eye movements. Our limited sample size for mouth movement

provided a large range for threshold choosing. Since 0.1 KS distance and the 400-pixel combination were the optimum combinations in this range, we also used them for mouth movement detection.

Video Recordings

All videos were recorded using a Canon Rebel SL3 at 29.97 frames per second. The camera focused on the patient's face for all videos, and adequate lighting was achieved. The camera was placed approximately six feet away from the patient's face and at the level of the patient's face. The head of the hospital bed was elevated to 30 degrees for all patients. Healthy subjects were studied with similar techniques.

Supplementary Results

Detecting Low-Amplitude Movements Beyond the Naked Eye

SeeMe was much more sensitive to low-amplitude movements than blinded raters watching the videos with the naked eye (Supplementary Figure 9). For eye movements, SeeMe detected nearly four times as many small movements whose amplitude was less than 2x threshold (movements < 800 pixels, 342 SeeMe-observed movements versus 85 blinded rater-observed movements. SeeMe detected approximately ~1.8 times as many low-amplitude mouth movements (movements < 800 pixels, 284 SeeMe-observed versus 157 blinded rater-observed (Supplementary Figure 9B). The number of SeeMe+ trials exceeds that of blinded raters across different eye and mouth video amplitude ranges, respectively. In particular, SeeMe detects far more responses in the low amplitude range than the blinded raters.

SeeMe Detects Movement Before Clinical Examiners in Most Patients

For each patient, the time until eye and mouth movement detection between SeeMe and clinical examiners using GCS and CRS-R, respectively, is compared (Supplementary Figure 10). Dots above the black dashed line mean SeeMe detected responses before the clinical examiners. Out of the seven patients detected with both clinical examiners using CRS-R auditory subscore and SeeMe mouth commands, five had detectable movements by SeeMe

before command following on clinical examination (5/7 patients, dots within the green triangle). There were five additional patients who SeeMe identified as following mouth commands, but clinical examinations did not detect command following during the study period. Of these patients, three went on to follow commands by discharge.

There were two patients who clinical examiners identified as command following before SeeMe. Upon inspecting the individual data for each patient, we found possible explanations for each. In one patient, the SeeMe study team did not pause sedation during the session, while the clinical examiners conducted their assessment with sedation paused. In the other patient, on review of the video recording of the CRS evaluation, it was independently determined that the CRS-R was incorrectly assessed by the study member at the time.

Post Hoc Analysis for Response Amplitude/Frequency and Discharge Outcome

The results of the post hoc pairwise comparisons analysis showed the significant correlation between GOS-E at discharge and SeeMe+ response amplitude contributed to the difference in amplitude between patients who died (GOS-E = 1) and those with severe disabilities (GOS-E = 3, p = 0.017) and moderate disability (GOS-E = 4, p = 0.001), and also between patients in the vegetative state (GOS-E = 2) and moderate disability (GOS-E = 4, p = 0.036). Moreover, the significant correlation between response detection frequency and GOSE at discharge contributed to the difference in response detection frequency between patients with the worst outcome (GOS-E = 1) and severe disabilities (GOS-E = 3, p = 0.018), and also between patients in the vegetative state (GOS-E 2) and severe disability (GOS-E = 3, p = 0.017).

SeeMe's Performance Correlates with Patients' 6-Month Outcomes

We also investigated the relationship between SeeMe performance and patients' functional outcomes as measured by GOS-E at 6 months. SeeMe performance was assessed using both SeeMe+ trial response amplitude and the response detection frequency (fraction of

SeeMe+ trials to the total number of trials). Both amplitude (N = 293, $w = 40.12$, $df = 3$, $p = 9.58 \times 10^{-9}$) and response detection frequency (N = 251, $w = 12.93$, $df = 3$, $p = 0.005$) correlated positively with six-month outcome (Supplementary Figure 4).

The post hoc pairwise comparisons showed the significant correlation between GOS-E at 6-months and response amplitude contributed to the differences in amplitude between patients with moderate disability (GOS-E = 4) and those who died (GOS-E = 1, $p = 1.83 \times 10^{-4}$), were in a vegetative state (GOS-E = 2, $p = 2.65 \times 10^{-5}$), or had severe disabilities (GOS-E = 3, $p = 9.95 \times 10^{-9}$). Also, the significant correlation between GOS-E at 6 months and response detection frequency contributed to the difference in response detection frequency between patients in the vegetative state (GOS-E 2) and moderate disability (GOS-E = 4, $p = 0.006$).

Sedation level and complications

Most (62.8%) videos were recorded while patients were not receiving any sedation. However, 9.8% of the videos were recorded while the patients were on continuous sedation infusion at a deep sedation dosage, and 27% were recorded under light sedation. For these patients, we attempted to pause sedation 15 to 30 minutes before the session whenever clinically feasible. Based on chi-square analysis, we demonstrated a significant association between the level of sedation and SeeMe results ($p = 1.54 \times 10^{-6}$). The proportion of light sedation was slightly lower in SeeMe+ videos (18.5%) compared to SeeMe- videos (25.2%), while deep sedation was much less common in SeeMe+ videos (3.5%) than in SeeMe- videos (16.3%) (Supplementary Table 1).

We reported the most common complications with potential influence on the level of consciousness within one week of incidence. We also found significant associations between SeeMe results and several complications. Hospital-acquired pneumonia showed an association ($p = 0.017$), while pulmonary embolism ($p = 2.29 \times 10^{-5}$) and delirium ($p = 6.57 \times 10^{-4}$) demonstrated significant associations with SeeMe results. The proportion of hospital-acquired

pneumonia and pulmonary embolism was higher in SeeMe- videos (14.1% and 11.1%) compared to
SeeMe+ videos (7% and 1.4%), while delirium status was more common in SeeMe+ videos (15.7%) than in SeeMe- videos (4.4%). Patients with pulmonary embolism required significantly more deep sedation compared to those without (31.6% in patients with pulmonary embolism group vs. 6.5%, $p = 5.31 \times 10^{-4}$). Similarly, patients with hospital-acquired pneumonia were more likely to require deep sedation (20.5% vs. 6.3%, $p = 3.95 \times 10^{-4}$). However, there was no significant association between delirium and sedation levels ($p = 0.724$).

Confusion Matrix

Using the optimally identified decision threshold (KS stats threshold = 0.1 and post peak threshold = 400), SeeMe demonstrated the following performance metrics when compared to blinded raters for “Open your eyes,” “Stick out your tongue,” and “Show me a smile” trials on all patients: sensitivity = 86.6% / 75.0%, specificity = 55.4% / 56.7%, the relatively low specificity is expected, as it likely reflects that SeeMe can detect responses in trials where blinded raters do not. Blinded raters were chosen for this comparison because they were the only metric that could be applied on the individual trial level (as opposed to GCS and CRS-R).

Supplementary Figures

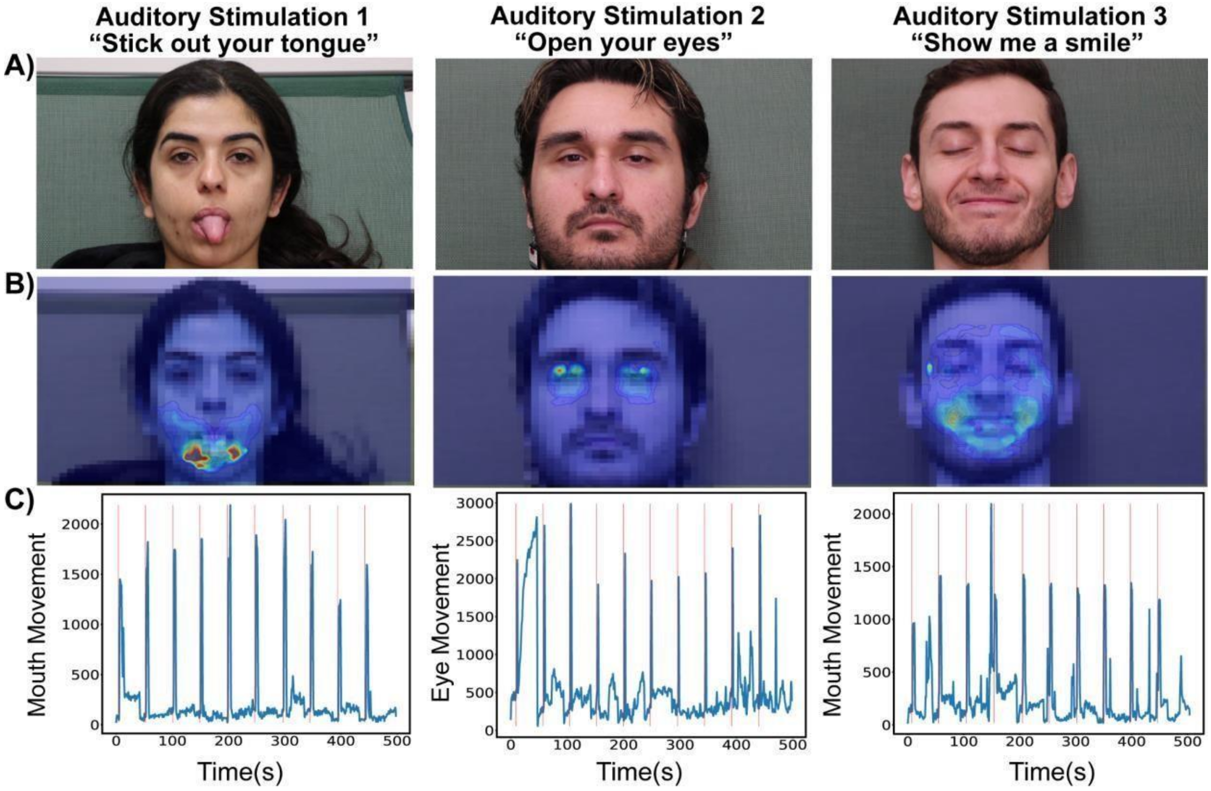

Supplementary Figure 1. Examples of SeeMe heatmaps and landmark summation plots in healthy control subjects. Here, we show examples where SeeMe detected stimulus-evoked movements in response to the auditory stimulation commands. **(A)** Example frames from healthy subjects who consented to show their faces in publication. One frame from each auditory stimulation type is shown. **(B)** Movement heatmaps for the corresponding frames are shown in **A**. **(C)** Landmark summation plots for video trials associated with the frames shown in **A** and **B**. Blue lines show the movement summation as the trial progressed. In contrast, the red vertical lines indicate the start of each auditory stimulation command. All individuals shown provided written informed consent for their images to be published.

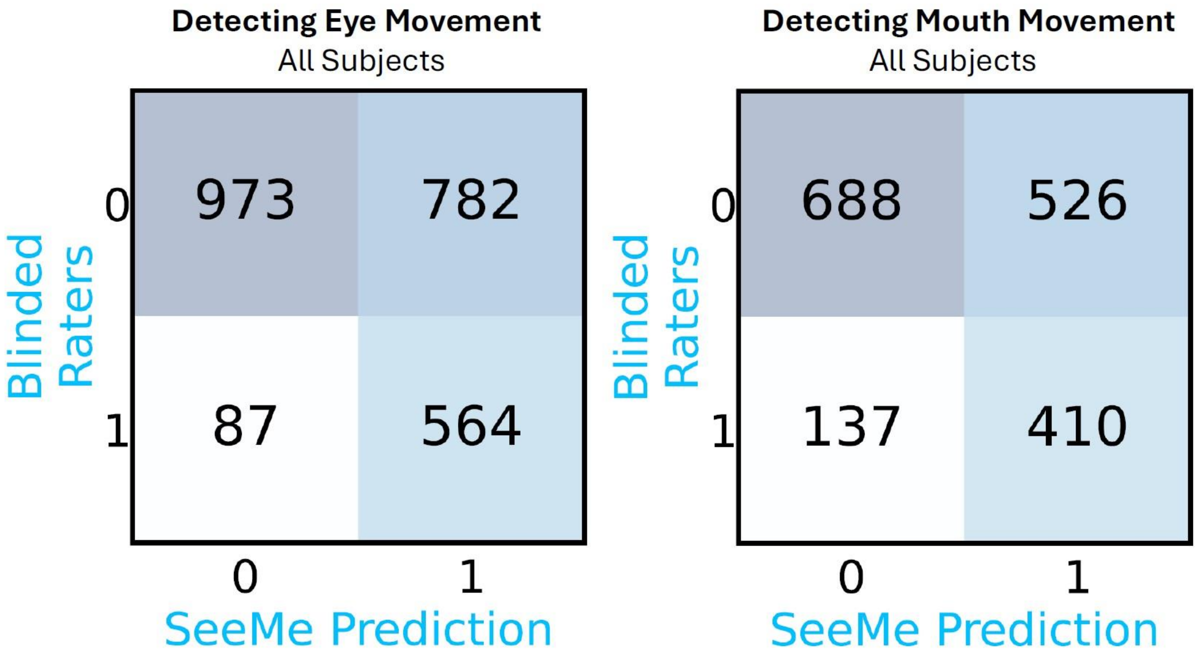

Supplementary Figure 2. Confusion matrices comparing SeeMe Eye and Mouth movement detection with blinded raters. Here, we compare the number of positive and negative responses determined by the blinded raters and SeeMe for all ABI Subjects. The *left* column pertains to the "Open your eyes" command, and the *right* pertains to the "Show me a smile" and "Stick out your tongue" commands together. Only clean videos were considered. For mouth commands, only the videos of the patients not wearing ventilation equipment were used.

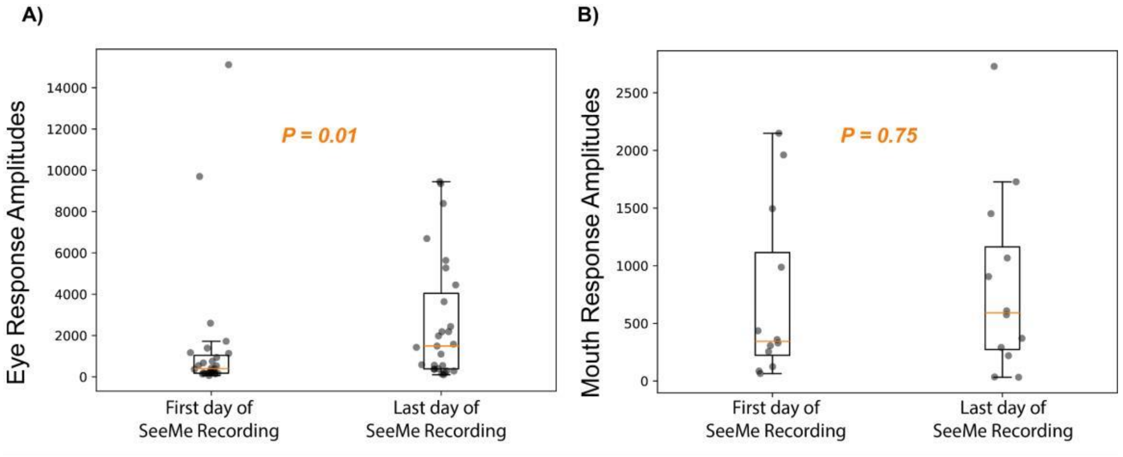

Supplementary Figure 3. SeeMe response amplitude progression over time. (A) Box plots comparing the average maximum (per trial) eye response per video for 30 ABI patients listening to Auditory Stimulation 2, “Open your eyes,” on the first and last days of SeeMe. The yellow line represents the median of each data set. **(B)** Box plots comparing the average maximum (per trial) mouth response per video for the 16 ABI patients listening to Auditory Stimulation 1, “Stick out your tongue,” and 3, “Show me a smile,” detected on SeeMe. The yellow line represents the median of each data set.

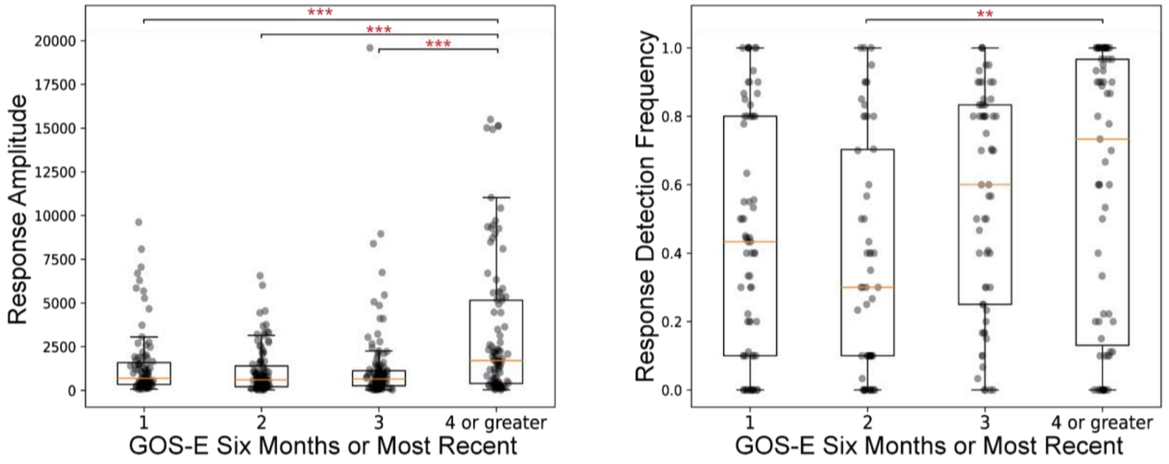

Supplementary Figure 4. SeeMe responses correlate with patients' outcomes at six months or at most recent assessment. Box plots comparing the average maximum amplitude **(A)** per SeeMe+ video and the average fraction of SeeMe+ trials **(B)** per video for all 36 ABI patients, based on the patient's GOS-E at six months or at the most recent assessment. The patients are divided into dead (GOS-E = 1), vegetative (GOS-E = 2), conscious severe disability (GOS-E = 3) and conscious moderate disability (GOS-E = 4). The yellow line represents the median of each data set. Kruskal-Wallis test followed by post-hoc pairwise comparisons using the Dunn-Bonferroni approach; *p < 0.05, **p < 0.01, ***p < 0.001.

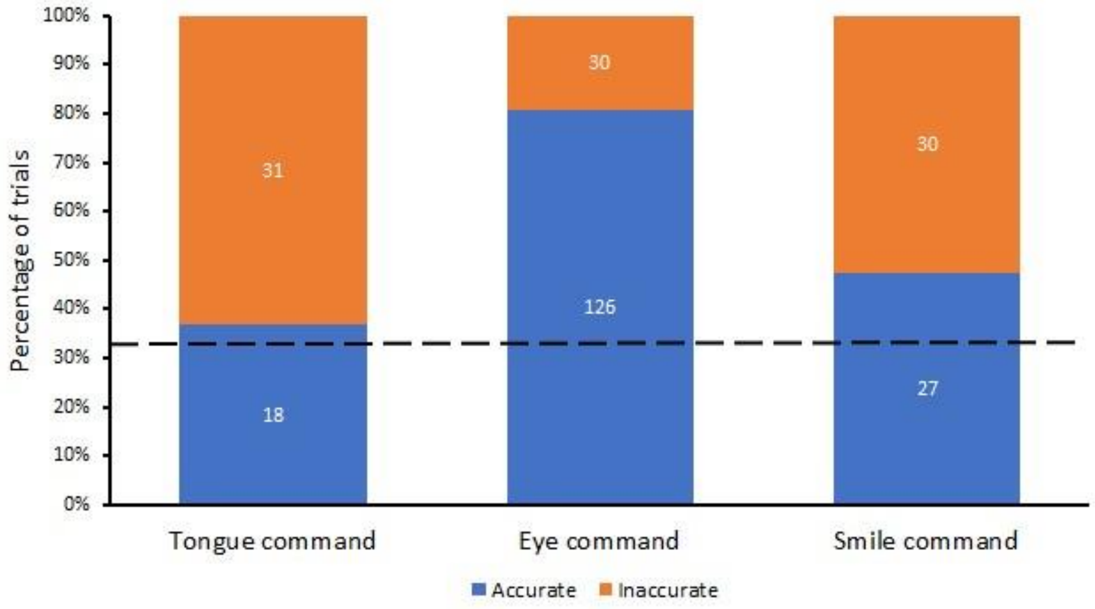

Supplementary Figure 5. SeeMe+ classifier performance. This figure shows the percentages of accurate and inaccurate predictions of our deep neural network classifier on the SeeMe+ testing dataset. Tongue command corresponds to the “Stick out your tongue” command, eye command corresponds to the “Open your eyes” command, and smile command corresponds to the “Show me a smile” command. The number of trials within each category are shown within each bar. The classifier’s performance exceeded chance accuracy (33%), indicated by the dashed line, and reached 81% accuracy for eye commands and 65% overall accuracy.

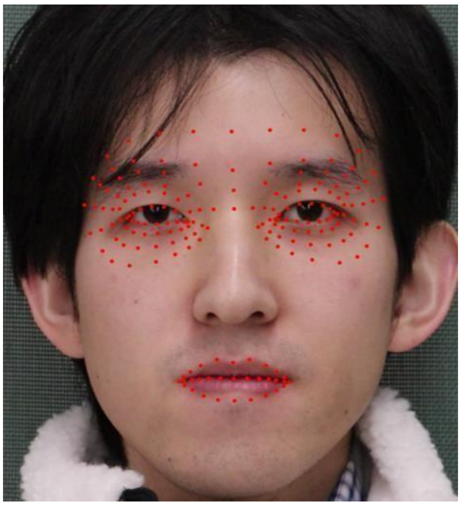

Supplementary Figure 6. The facial landmarks of a healthy subject. This is an example of facial landmark identification and region of interest definition for the eye and mouth areas of a healthy subject. The individual shown provided written informed consent for their image to be published.

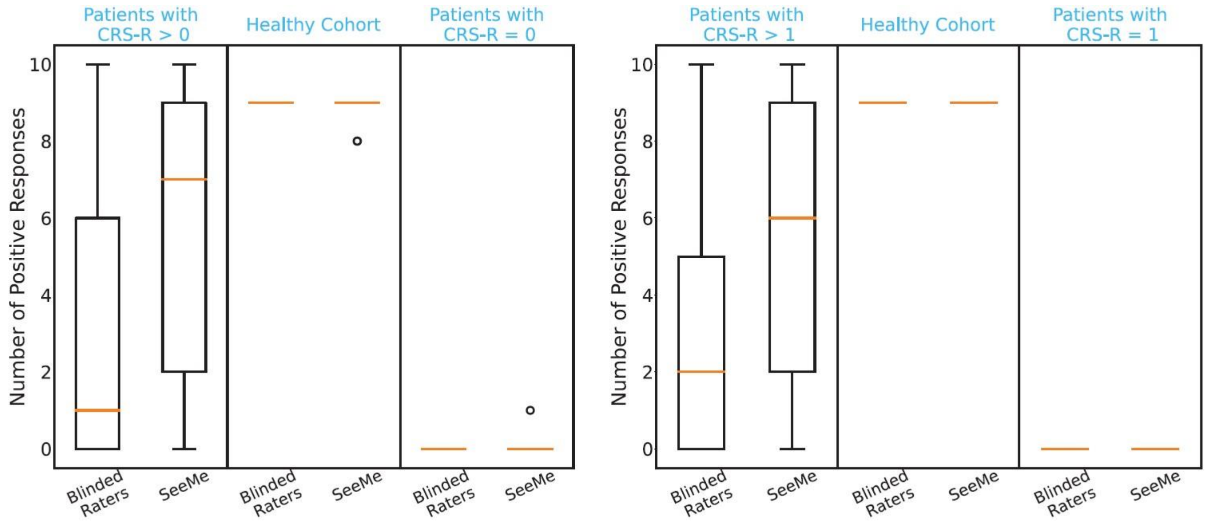

Supplementary Figure 7. SeeMe eye and mouth responses in healthy control and comatose ABI cohorts. Box plots demonstrating the median number of positive responses per video (maximum 10) determined by blinded raters and SeeMe across different subject cohorts in response to eye (*left*) and mouth commands (*right*). For each box plot, the *left* panel uses comatose patients whose CRS-R score was > 0 and > 1 respectively.. The *middle* panel uses healthy subjects, and the *right* uses comatose patients with CRS-R = 0. For mouth commands, the *right* third uses comatose patients with CRS-R = 1 (no videos from patients with CRS-R = 0 without ventilation equipment). The red line represents the median, and the box represents 25%-75% of the data.

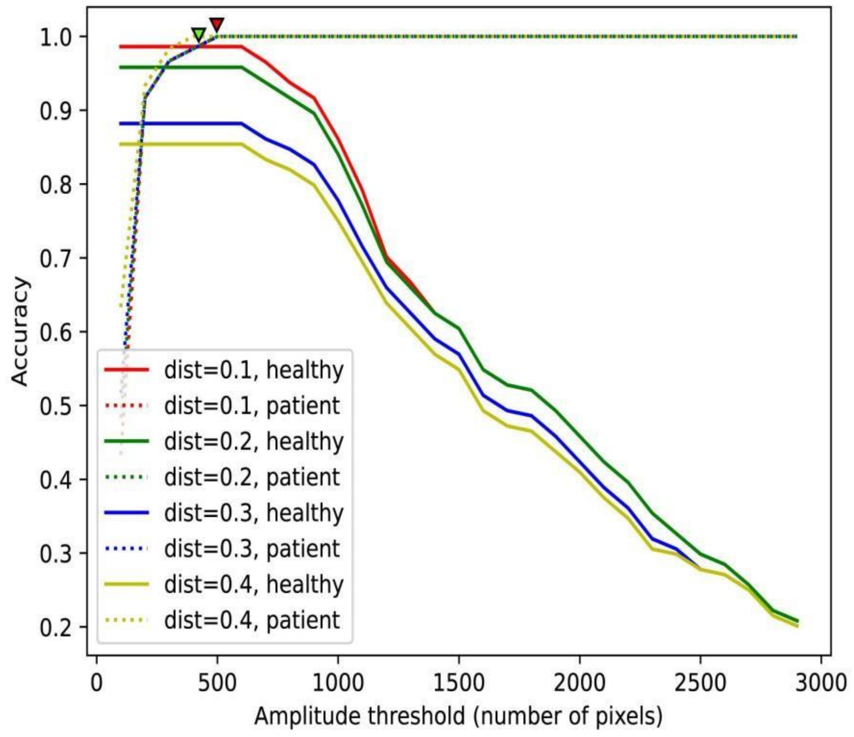

Supplementary Figure 8. Determination of sensitivity thresholds for SeeMe. SeeMe only identifies responses as significant if their KS distance is larger than a distance threshold and their maximum response amplitude is larger than the amplitude threshold. If the distance and amplitude thresholds are too large, SeeMe may miss responses. If they are too small, SeeMe may treat some spurious movements as responses. We determine the two thresholds by maximizing the prediction accuracy for healthy controls (SeeMe should be able to detect responses in all their trials) and deeply comatose patients (SeeMe shouldn't detect any responses in all their trials). Here, we plot SeeMe prediction accuracy for deeply comatose and healthy subjects using different threshold combinations in two groups. The solid and dashed lines with the same color represent SeeMe prediction accuracy for healthy controls and deeply comatose patients using the same distance threshold. We want to find a threshold combination such that the solid and dashed lines with the same color are high in that position. From the figure, we can see using a distance threshold of 0.1 and an amplitude threshold of 500 (red triangle) is the optimum. We chose a distance threshold of 0.1 and an amplitude threshold of

400 (green triangle) to have nearly 100% accuracy in healthy and motionless patients and capture weak responses in ABI patients.

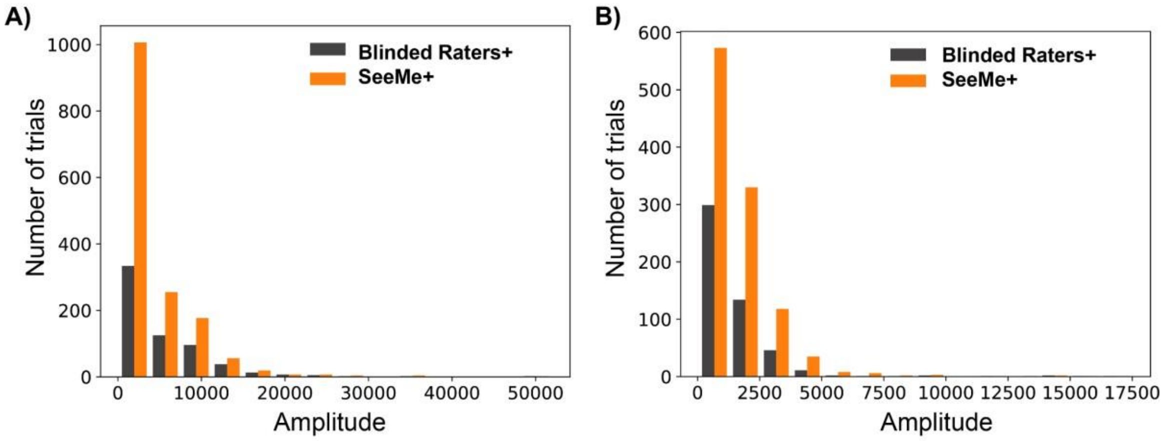

Supplementary Figure 9. SeeMe detection of low-amplitude movements invisible to the naked eye. (A) Histogram showing the amplitude distribution of SeeMe+ trials versus the amplitude distribution of the blinded raters' positive trials from 36 ABI patients listening to Auditory Stimulation 2, "Open your eyes." Orange represents SeeMe's positive trials, and blue represents the blinded raters' positive trials. The mean amplitude of eye movements increased from 1446 pixels ± 3233 (mean ± SD) on the first day of SeeMe (mean days 9.1 ± 5.3 SD after admission) to 2634 pixels ± 2896 on the last day of SeeMe (mean days 23.6 ± 15.4 SD after admission, Mann-Whitney U test p = 0.01). **(B)** Histogram showing the amplitude distribution of SeeMe+ trials versus the amplitude distribution of the blinded raters' positive trials across 17 ABI patients listening to Auditory Stimulation 1, "Stick out your tongue," and 3, "Show me a smile." The mean amplitude of mouth movements increased from 712 pixels ± 718 on the first day (mean days 19.1 ± 10.2 SD after admission) to 834 pixels ± 770 on the last day (mean days 31.1 ± 14.5 SD after admission, p = 0.75). Average amplitudes for each patient are reported in (Supplementary Table 3).

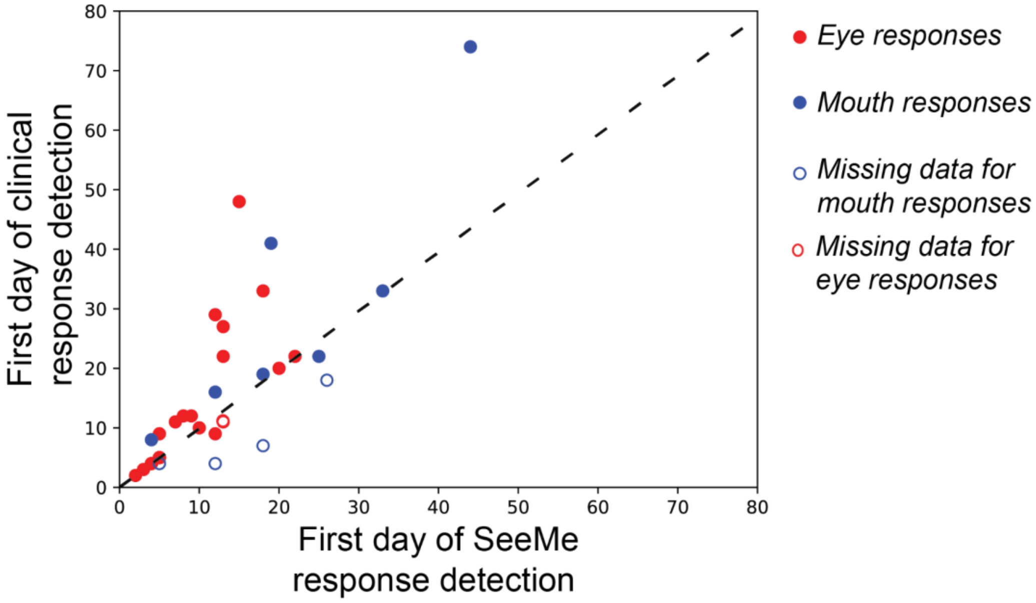

Supplementary Figure 10. Detection of responses by SeeMe and clinical examiners. This graph illustrates the days for SeeMe and clinical examiners to detect eye (red) and mouth (blue) responses. The x-axis represents the first day a patient has a SeeMe+ video, and the yaxis represents the first day the clinical examiner detected responses. The one red circle and four blue circles represent patients with no analyzable eye/mouth command video (patients with ventilators obstructing their mouth region) before/on the day the clinical examiner detected the command following. The patient corresponding to the only red dot representing clinical response detection earlier than SeeMe was on deep sedation on the SeeMe test.

Supplementary Table 1. Level of sedation, complications, and SeeMe results. The level of sedation is categorized as no sedation, light, or deep sedation, based on the dosage of medications. We reported the complications that co-occurred within one week of individual SeeMe sessions that could influence the level of consciousness. A chi-square test was used to evaluate the relationship between level of sedation or complications and SeeMe results.

**SeeMe prediction**		**Positive**	**Negative**	**P-value**
**Number of SeeMe videos**		287	135	
**Level of Sedation**	No	78.0%	58.5%	**1.54×10^{-6}***
	Light	18.5%	25.2%	
	Deep	3.5%	16.3%	
**Stroke**	Yes	3.8%	3.7%	0.594
	No	96.2%	96.3%	
**ICH**	Yes	3.5%	1.5%	0.204
	No	96.5%	98.5%	
**Convulsions**	Yes	1.7%	0.7%	0.377
	No	98.3%	99.3%	
**Neurological infections**	Yes	-	-	-
	No	100%	100%	
**CSF Leak**	Yes	0.7%	1.5%	0.081
	No	99.3%	98.5%	
**Hydrocephalus**	Yes	9.1%	8.1%	0.458
	No	90.9%	91.9%	
**Cerebral edema**	Yes	3.1%	6.7%	0.383
	No	96.9%	93.3%	
**Hospital-acquired pneumonia**	Yes	7.0%	14.1%	**0.017***
	No	93.0%	85.9%	
**Aspiration pneumonia**	Yes	5.6%	5.2%	0.536
	No	94.4%	94.8%	
**Pulmonary Embolism**	Yes	1.4%	11.1%	**2.29×10^{-5}***
	No	98.6%	88.9%	
**Sepsis**	Yes	2.4%	3%	0.489
	No	97.6%	97%	
**Acute renal failure**	Yes	1.4%	0.7%	0.487
	No	98.6%	99.3%	
**Delirium**	Yes	15.7%	4.4%	**6.57×10^{-4}***
	No	84.3%	95.6%	

*$p < 0.05$.

Supplementary Table 2. Comatose patient outcomes and consciousness detection timelines via SeeMe and clinical examination. This table depicts each ABI patient, their clinical outcomes, and the first-day SeeMe and clinical examiners (GCS eyeopening subscore > 2 and CRS-R auditory subscore >2) detected stimulus-evoked facial movements. Patients detected via SeeMe for mouth and eye movements before clinical observers are highlighted in purple. Those with only eye movements detected by SeeMe before clinical examiners are highlighted in red. For patients that went undetected by CRS-R during the study but later went on to follow commands (first day of two continuous GCS motor subscore = 6 after first available SeeMe video), their day of command following was placed in parentheses.

Subject Number	Age and Sex	Mechanism of injury	Initial CRSR	Final CRS-R	Discharge GCS	Discharge GOS-E	FC at discharge	Follow up 6 Month GOS-E	1st Day of SeeMe Detected Eye Movement	1st Day of Responsiveness via GCS (Eye opening subscore > 2)	1st Day of SeeMe Detected Mouth Movement	1st Day of Responsiveness via CRS-R (Auditory subscore > 2)
2	50-59 M	TBI	1	16	11	3	Yes	3	13	22	19	41
3	50-59 M	Aneurysmal SAH	1	13	11	3	Yes	1	15	48	48	CRS-R - (40)
9	60-79 F	TBI	2	12	10	3	Yes	2	5	9	13	CRS-R - (44)
12	30-39 M	TBI	0	19	15	3	Yes	4	8	12	18	19
13	60-69 M	TBI	1	8	12	3	Yes	3	10	12	18	CRS-R - (20)
16	50-59 M	TBI	3	16	10	2	No	2	12	29	44	74
24	60-69 M	TBI	6	6	7	2	No	-	6	GCS -	6	CRS-R -
36	60-69 M	ICH	2	2	10	2	No	-	3	GCS-	20	CRS-R -
1	70-79 F	Spontaneous ICH	1	4	3	1	No	1	12	GCS -	NA	CRS-R -
5	50-59 F	Meningo-encephalitis	2	2	4	1	No	1	10	GCS -	NA	CRS-R -
11	60-69 F	TBI	2	1	5	1	No	1	5	GCS -	NA	CRS-R -
17	50-59 M	TBI	7	8	7	3	Yes	3	18	33	NA	CRS-R - (108)
19	50-59 M	TBI	0	21	12	3	Yes	4	13	27	33	33
23	40-49 M	ICH	6	6	9	2	No	-	14	GCS -	NA	CRS-R - (29)
30	50-59 F	TBI	3	11	3	1	No	1	7	11	NA	12
31	50-59 M	ICH	0	13	6	3	Yes	-	22	23	25	22
4	80-89 M	TBI	4	13	11	3	Yes	1	4	4	18	7
6	60-69 M	TBI	16	23	15	4	Yes	8	5	5	12	4
7	20-29 M	TBI	4	22	15	4	Yes	5	12	9	12	16
8	60-69 M	TBI	6	14	10	3	Yes	1	4	4	4	8
10	60-69 M	Spontaneous ICH	1	1	10	3	Yes	2	SeeMe -	GCS -	SeeMe -	CRS-R - (27)
14	10-19 M	TBI	0	7	15	3	Yes	8	SeeMe -	GCS -	NA	CRS-R - (28)
15	60-69 M	TBI	3	6	7	1	No	1	20	20	NA	CRS-R -
18	30-39 M	TBI	6	19	15	3	Yes	3	2	2	NA	8
20	70-79 F	TBI	11	2	3	1	No	1	10	10	NA	15
21	40-49 M	TBI	5	15	14	4	Yes	-	5	5	NA	8
22	80-89 M	TBI	22	22	15	3	Yes	-	3	3	NA	3
25	20-29 M	TBI	12	14	15	4	Yes	-	5	5	NA	11
26	50-59 M	TBI	0	14	14	4	Yes	-	5	5	NA	CRS-R-
27	20-29 M	TBI	11	11	15	4	Yes	-	SeeMe -	14	NA	CRS-R - (18)
28	40-49 F	ICH	1	1	3	1	No	1	SeeMe -	GCS -	NA	CRS-R -
29	70-79 F	Hydrocephalus	17	9	10	3	Yes	-	4	4	5	4
32	70-79 M	ICH	10	7	7	2	No	-	SeeMe-	GCS-	NA	CRS-R -
33	40-49 M	TBI	6	17	15	4	Yes	-	13	11	26	18
34	30-39 M	TBI	9	12	15	4	Yes	-	5	5	NA	CRS-R - (6)
35	30-39 M	TBI	8	18	15	3	Yes	-	SeeMe-	2	NA	4
37	20-29 M	TBI	2	21	15	3	Yes	-	NA	6	NA	6

M, male; F, female; TBI, traumatic brain injury; ICH, intracerebral hemorrhage; GOS-E, Glasgow Outcome Scale–Extended; GCS, Glasgow Coma Scale; CRS-R, Coma Recovery Scale–Revised; NA, no available video

Supplementary Table 3. SeeMe pixel amplitude progression over time. For patients who had SeeMe+ videos, we listed the days with SeeMe+ videos and SeeMe response amplitude (the average and standard deviation) for SeeMe+ videos. The last column is the mean amplitude of SeeMe on the day the clinical examinations (CRS-R/GCS) detected responsiveness. The first table corresponds to eye commands, and the second corresponds to mouth commands. NA means either the patient is never detected responsive by a clinical examiner or not detected by a clinical examiner on the days they have SeeMe+ videos.

Subject Number	Days with SeeMe+ Videos	Amplitude on First Day of SeeMe+ Video	Amplitude on Last Day of SeeMe+ Video	Mean Amplitude of SeeMe-detected Movements (± Standard Deviation)	Mean Amplitude of SeeMe-detected Movements on Clinical Response Days (± Standard Deviation)
2	15	416.42	6743.09	4507.95 ± 4843.33	5043.01 ± 4989.87
3	2	438.83	5853.77	3146.3 ± 2707.47	5853.77
4	14	2596.75	5685.49	3748.03 ± 2558.0	3663.69 ± 2491.33
6	4	15115.6	1583.17	8050.28 ± 5325.01	8050.28 ± 5325.01
7	6	3140.34	1982.88	7206.75 ± 4496.18	4878.22 ± 4931.81
8	8	1721.74	1491.39	1457.03 ± 364.96	1457.03 ± 364.96
9	7	547.79	586.77	1967.11 ± 2062.84	1648.11 ± 2174.96
12	9	5587.28	3939.32	5152.26 ± 1990.87	5060.91 ± 2248.26
13	10	832.95	2189.09	1750.5 ± 1284.98	1653.3 ± 1505.04
15	2	618.32	394.87	506.6 ± 111.73	506.6 ± 111.73
16	22	2549.23	6015.65	2521.72 ± 1455.31	2885.62 ± 1468.42
17	4	1135.94	564.05	1481.73 ± 816.83	NA
18	4	937.42	1098.41	2204.47 ± 1292.84	2204.47 ± 1292.84
19	11	388.43	14932.69	8149.18 ± 5282.62	11058.29 ± 3626.86
20	7	1170.48	1402.75	940.68 ± 318.7	1024.33 ± 230.0
21	3	1241.75	9454.73	3742.59 ± 4049.49	3742.59 ± 4049.49
22	1	5064.38	5064.38	5064.38	5064.38
25	2	9705.35	10428.5	10066.92 ± 361.57	5172.35 ± 4901.24
26	2	2162.8	5637.55	3900.18 ± 1737.38	3900.18 ± 1737.38
29	3	751.51	2435.24	1677.66 ± 697.64	1677.66 ± 697.64
30	3	361.06	5273.84	5085.3 ± 3782.71	7447.42 ± 2173.58
31	9	494.17	1322.2	678.63 ± 298.89	552.35 ± 340.97
33	3	364.75	4450.26	3838.62 ± 2622.61	5575.56 ± 1125.3
34	2	1389.73	546.2	967.96 ± 421.76	967.96 ± 421.76

Subject Number	Days with SeeMe+ videos	Amplitude on First Day of SeeMe+ Video	Amplitude on Last Day of SeeMe+ Video	Mean Amplitude of SeeMe-detected Movements (± Standard Deviation)	Mean Amplitude of SeeMe-detected Movements on Clinical Response Days (± Standard Deviation)
2	27	389.16	1008.85	889.25 ± 298.83	894.36
4	16	1960.7	1651.81	1645.76 ± 595.25	2391.07 ± 352.15
6	4	987.26	574.57	780.92 ± 263.03	780.92 ± 263.03
7	6	1592.69	1451.52	1959.14 ± 759.38	1829.46 ± 768.82
8	3	359.22	291.24	408.82 ± 74.91	239.31 ± 87.52
12	8	2148.87	1727.95	1617.95 ± 371.8	1542.1 ± 334.61
16	17	213.09	1544.6	891.83 ± 328.61	NA
19	11	1687.81	2286.34	2505.92 ± 1030.57	2675.1 ± 1067.38
29	3	436.01	608.91	552.7 ± 114.14	NA
31	4	297.55	395.25	396.52 ± 20.55	228.15 ± 115.92
33	2	307.05	219.55	442.18 ± 37.2	536.65 ± 473.31

References

1. Lugaresi C, Tang J, Nash H, et al. MediaPipe: A Framework for Building Perception Pipelines. *arXiv.org*. Published online 2019. Accessed May 8, 2023. https://www.semanticscholar.org/paper/1c44a3cde07f3f2ffd8880b441449483ed74fc5f

2. Peters WH, Ranson WF. Digital Imaging Techniques In Experimental Stress Analysis. *Organ Ethic*. 1982;21(3):427-431. doi:10.1117/12.7972925
