# Supplementary material for: Computer vision detects covert voluntary facial movements in unresponsive brain injury patients
Source: Commun Med (Lond). 2025 Aug 20;5:361. doi: 10.1038/s43856-025-01042-y (PMC12368019; doi:10.1038/s43856-025-01042-y)
Supplement: Supplementary file 3 — Description of Additional Supplementary Files [file 43856_2025_1042_MOESM3_ESM.pdf]

Description of Additional Supplementary Files

1- File name: Supplementary Video 1
File description: **Subject 13 Auditory Stimulation 2.** An example of a detected response to Auditory Stimulation 2, “Open your eyes.”

2- File name: Supplementary Video 2
File description: **Subject 2 Auditory Stimulation 3.** An example of a detected response to Auditory Stimulation 3, “Show me a smile.”

3- File name: Supplementary Data 1.xlsx
File description: Source data for Table 1, Figure 4a, Figure 4b, Supplementary Figure 4a, Supplementary Figure 4b, and Supplementary Table 1. Includes all numerical values used to generate the figures and table content.
