# Supplementary material for: Computer vision detects covert voluntary facial movements in unresponsive brain injury patients
Source: Commun Med (Lond). 2025 Aug 20;5:361. doi: 10.1038/s43856-025-01042-y (PMC12368019; doi:10.1038/s43856-025-01042-y)
Supplement: Supplementary file 7 — Reporting Summary [file 43856_2025_1042_MOESM7_ESM.pdf]

nature portfolio

Corresponding author(s): Dr. Sima Mofakham

Last updated by author(s): June 14, 2025

Reporting Summary

Nature Portfolio wishes to improve the reproducibility of the work that we publish. This form provides structure for consistency and transparency in reporting. For further information on Nature Portfolio policies, see our Editorial Policies and the Editorial Policy Checklist.

Please do not complete any field with "not applicable" or n/a. Refer to the help text for what text to use if an item is not relevant to your study.
For final submission: please carefully check your responses for accuracy; you will not be able to make changes later.

Statistics

For all statistical analyses, confirm that the following items are present in the figure legend, table legend, main text, or Methods section.

n/a	Confirmed	
☐	☑	The exact sample size (*n*) for each experimental group/condition, given as a discrete number and unit of measurement
☐	☑	A statement on whether measurements were taken from distinct samples or whether the same sample was measured repeatedly
☐	☑	The statistical test(s) used AND whether they are one- or two-sided<br>*Only common tests should be described solely by name; describe more complex techniques in the Methods section.*
☐	☑	A description of all covariates tested
☐	☑	A description of any assumptions or corrections, such as tests of normality and adjustment for multiple comparisons
☐	☑	A full description of the statistical parameters including central tendency (e.g. means) or other basic estimates (e.g. regression coefficient) AND variation (e.g. standard deviation) or associated estimates of uncertainty (e.g. confidence intervals)
☐	☑	For null hypothesis testing, the test statistic (e.g. *F*, *t*, *r*) with confidence intervals, effect sizes, degrees of freedom and *P* value noted<br>*Give P values as exact values whenever suitable.*
☒	☐	For Bayesian analysis, information on the choice of priors and Markov chain Monte Carlo settings
☒	☐	For hierarchical and complex designs, identification of the appropriate level for tests and full reporting of outcomes
☒	☐	Estimates of effect sizes (e.g. Cohen's *d*, Pearson's *r*), indicating how they were calculated

Our web collection on statistics for biologists contains articles on many of the points above.

Software and code

Policy information about availability of computer code

Data collection: No software was used for data collection.

Data analysis:
1. The Python-based video processing pipeline described in this study was conducted using Python V3.12.
2. Facial landmark generation using these videos utilized the publicly available Mediapipe machine learning algorithm.
3. All analyses were conducted using either Python V3.12 or IBM-SPSS Statistics V29.

For manuscripts utilizing custom algorithms or software that are central to the research but not yet described in published literature, software must be made available to editors and reviewers. We strongly encourage code deposition in a community repository (e.g. GitHub). See the Nature Portfolio guidelines for submitting code & software for further information.

Data

Policy information about availability of data

All manuscripts must include a data availability statement. This statement should provide the following information, where applicable:

- Accession codes, unique identifiers, or web links for publicly available datasets
- A description of any restrictions on data availability
- For clinical datasets or third party data, please ensure that the statement adheres to our policy

All data reported in Table 1, Figure 4A/4B, and Supplementary Table 1 can be found in the attached Supporting Data Values file. The remaining data that support the findings of this study are available on request from the corresponding author. Video data are not publicly available due to privacy or ethical restrictions.

Research involving human participants, their data, or biological material

Policy information about studies with human participants or human data. See also policy information about sex, gender (identity/presentation), and sexual orientation and race, ethnicity and racism.

Reporting on sex and gender	This study reported sex as one of its demographic characteristics. Healthy participants self-reported this data. Information for comatose patients was obtained from the hospital EMR. Typically, this information is inpuuted by hospital staff, but can be updated with additional info from NOK. Consent obtained for disclosure of this info from all participants and their LARs. The total study population (N = 53) had a median age of 44 and predominantly consisted of males (N = 40). Of this cohort, the healthy population (N = 16) had a median age of 27 with 11 males, 5 females. The comatose cohort (N = 37) had a median age of 57 and were predominantly males (N = 29). All participants were consecutively enrolled following injury. As males are more likely to experience the injuries described in this study, the composition of the comatose may reflect this predisposition. Given the majority of our comatose cohort consisted of males, sex-based analyses were not performed.
Reporting on race, ethnicity, or other socially relevant groupings	Race and ethnicity were reported for this study. Healthy participants self-reported this data, while information for comatose patients was obtained from the hospital electronic medical record. Typically, this information is inputted by hospital staff when the information is available, but can be updated by next-of-kin. Due to the nature of our consecutive enrollment process, our comatose cohort consisted predominantly of non-Hispanic (N = 27), White (N = 28) individuals. The healthy cohort consisted predominantly of non-Hispanic (N = 14), White (N = 13) individuals.
Population characteristics	Covariates assessed included sedation medications used, dosages, and complications that occurred during hospital stay that could have affected neurological status or session performance. Per video, sedation was classified as either heavy or light based on previous definitions established in the literature. The majority of sessions were performed with no sedation. Complication data collected included common complications faced by acute brain injury patients in intensive care units.
Recruitment	All participants were consecutively enrolled following injury. As males are more likely to experience the injuries described in this study, the composition of the comatose may reflect this predisposition.
Ethics oversight	Study approval was obtained from the Stony Brook University Institutional Review Board (IRB2019-00199).

Note that full information on the approval of the study protocol must also be provided in the manuscript.

Field-specific reporting

Please select the one below that is the best fit for your research. If you are not sure, read the appropriate sections before making your selection.

☑ Life sciences ☐ Behavioural & social sciences ☐ Ecological, evolutionary & environmental sciences

For a reference copy of the document with all sections, see nature.com/documents/nr-reporting-summary-flat.pdf

Life sciences study design

All studies must disclose on these points even when the disclosure is negative.

Sample size	Given the novelty of the methodology described in this report, estimates of effect size were not available at the initiation of the study.
Data exclusions	Video recordings were excluded in the presence of any artifact. Artifact was defined as any external influence that could have been falsely interpreted as movement from the patient themselves. For this study, we defined artifact as external movements of the patient from nursing staff or loved ones, movement of the camera during recording, and lighting changes. Additionally, for mouth commands, videos were excluded if patients were intubated due to limitations in generation of landmarks for the mouth.
Replication	The study protocol is described in detail in the manuscript and supplemental data files. Further information about study protocol can be obtained from the corresponding author.
Randomization	Due to the observational nature of this study, no randomization occurred.
Blinding	Blinding occurred at three levels for this study. Study team members who reviewed the session video recordings were blinded to the patients' clinical assessments, prognoses, and SeeMe results. Clinicians who were independent from the study team provided Glasgow Coma Scale (GCS) scores and were blinded to the SeeMe results. Coma Recovery Scale - Revised (CRS-R) scores were provided by study team members on the day of but prior to SeeMe session start and were blinded to the SeeMe results for each patient.

Behavioural & social sciences study design

All studies must disclose on these points even when the disclosure is negative.

Study description	
Research sample	
Sampling strategy	
Data collection	
Timing	
Data exclusions	
Non-participation	
Randomization	

Ecological, evolutionary & environmental sciences study design

All studies must disclose on these points even when the disclosure is negative.

Study description	
Research sample	
Sampling strategy	
Data collection	
Timing and spatial scale	
Data exclusions	
Reproducibility	
Randomization	
Blinding	

Did the study involve field work? ☐ Yes ☐ No

Field work, collection and transport

Field conditions	
Location	
Access & import/export	
Disturbance	

Reporting for specific materials, systems and methods

We require information from authors about some types of materials, experimental systems and methods used in many studies. Here, indicate whether each material, system or method listed is relevant to your study. If you are not sure if a list item applies to your research, read the appropriate section before selecting a response.

Materials & experimental systems

n/a	Involved in the study
X	☐ Antibodies
X	☐ Eukaryotic cell lines
X	☐ Palaeontology and archaeology
X	☐ Animals and other organisms
☐	✓ Clinical data
X	☐ Dual use research of concern
X	☐ Plants

Methods

n/a	Involved in the study
X	☐ ChIP-seq
X	☐ Flow cytometry
X	☐ MRI-based neuroimaging

Antibodies

Antibodies used	
Validation	

Eukaryotic cell lines

Policy information about cell lines and Sex and Gender in Research

Cell line source(s)

Authentication

Mycoplasma contamination

Commonly misidentified lines
(See ICLAC register)

Palaeontology and Archaeology

Specimen provenance

Specimen deposition

Dating methods

☐ Tick this box to confirm that the raw and calibrated dates are available in the paper or in Supplementary Information.

Ethics oversight

Note that full information on the approval of the study protocol must also be provided in the manuscript.

Animals and other research organisms

Policy information about studies involving animals; ARRIVE guidelines recommended for reporting animal research, and Sex and Gender in Research

Laboratory animals

Wild animals

Reporting on sex

Field-collected samples

Ethics oversight

Note that full information on the approval of the study protocol must also be provided in the manuscript.

Clinical data

Policy information about clinical studies
All manuscripts should comply with the ICMJE guidelines for publication of clinical research and a completed CONSORT checklist must be included with all submissions.

Clinical trial registration: This study is registered on ClinicalTrials.gov (SeeMe: An Automated Tool to Detect Early Recovery After Brain Injury, NCT06083441, Enrollment June 2019 - June 2025).

Study protocol: The study protocol is provided in the provided Manuscript and Supplemental Material files. Further information about the study can be requested from the corresponding author.

Data collection: Recruitment and data collection occurred between June 25, 2021, to August 27, 2024. Healthy controls performed study procedures in a private research space in the Department of Neurosurgery in Stony Brook University Hospital. Comatose patients participated in research procedures in intensive care units in Stony Brook University Hospital.

Outcomes: Primary outcomes of interest included detection of region-specific movements in response to commands as well as the timing of the detection of these movements in comparison with standard assessments of consciousness such as the Glasgow Coma Scale (GCS) and Coma Recovery Scale - Revised (CRS-R). Secondary outcomes included outcomes at discharge including Glasgow Outcome Scale - Extended (GOS - E) scores. These scores were used to assess if SeeMe results had prognostic implications for patients with acute brain injury.

Dual use research of concern

Policy information about dual use research of concern

Hazards

Could the accidental, deliberate or reckless misuse of agents or technologies generated in the work, or the application of information presented in the manuscript, pose a threat to:

No | Yes

- [ ] [ ] Public health
- [ ] [ ] National security
- [ ] [ ] Crops and/or livestock
- [ ] [ ] Ecosystems
- [ ] [ ] Any other significant area

Experiments of concern

Does the work involve any of these experiments of concern:

No | Yes

- [ ] [ ] Demonstrate how to render a vaccine ineffective
- [ ] [ ] Confer resistance to therapeutically useful antibiotics or antiviral agents
- [ ] [ ] Enhance the virulence of a pathogen or render a nonpathogen virulent
- [ ] [ ] Increase transmissibility of a pathogen
- [ ] [ ] Alter the host range of a pathogen
- [ ] [ ] Enable evasion of diagnostic/detection modalities
- [ ] [ ] Enable the weaponization of a biological agent or toxin
- [ ] [ ] Any other potentially harmful combination of experiments and agents

Plants

Seed stocks

Novel plant genotypes

Authentication

ChIP-seq

Data deposition

- [ ] Confirm that both raw and final processed data have been deposited in a public database such as GEO.
- [ ] Confirm that you have deposited or provided access to graph files (e.g. BED files) for the called peaks.

Data access links
May remain private before publication.

Files in database submission

Genome browser session
(e.g. UCSC)

Methodology

Replicates

Sequencing depth

Antibodies

Peak calling parameters

Data quality

Software

Flow Cytometry

Plots

Confirm that:

- [ ] The axis labels state the marker and fluorochrome used (e.g. CD4-FITC).
- [ ] The axis scales are clearly visible. Include numbers along axes only for bottom left plot of group (a 'group' is an analysis of identical markers).
- [ ] All plots are contour plots with outliers or pseudocolor plots.
- [ ] A numerical value for number of cells or percentage (with statistics) is provided.

Methodology

Sample preparation

Instrument

Software

Cell population abundance

Gating strategy

- [ ] Tick this box to confirm that a figure exemplifying the gating strategy is provided in the Supplementary Information.

Magnetic resonance imaging

Experimental design

Design type

Design specifications

Behavioral performance measures

Imaging type(s)

Field strength

Sequence & imaging parameters

Area of acquisition

Diffusion MRI [ ] Used [ ] Not used

Preprocessing

Preprocessing software

Normalization

Normalization template

Noise and artifact removal

Volume censoring

Statistical modeling & inference

Model type and settings

Effect(s) tested

Specify type of analysis: ☐ Whole brain ☐ ROI-based ☐ Both

Statistic type for inference

(See Eklund et al. 2016)

Correction

Models & analysis

n/a	Involved in the study
☐	☐ Functional and/or effective connectivity
☐	☐ Graph analysis
☐	☐ Multivariate modeling or predictive analysis

Functional and/or effective connectivity

Graph analysis

Multivariate modeling and predictive analysis

This checklist template is licensed under a Creative Commons Attribution 4.0 International License, which permits use, sharing, adaptation, distribution and reproduction in any medium or format, as long as you give appropriate credit to the original author(s) and the source, provide a link to the Creative Commons license, and indicate if changes were made. The images or other third party material in this article are included in the article's Creative Commons license, unless indicated otherwise in a credit line to the material. If material is not included in the article's Creative Commons license and your intended use is not permitted by statutory regulation or exceeds the permitted use, you will need to obtain permission directly from the copyright holder. To view a copy of this license, visit http://creativecommons.org/licenses/by/4.0/
